# Supplementary material for: Expanding the Chemical Space of Arsenicin A-C Related Polyarsenicals and Evaluation of Some Analogs as Inhibitors of Glioblastoma Stem Cell Growth
Source: Mar Drugs. 2023 Mar 17;21(3):186. doi: 10.3390/md21030186 (PMC10051910; doi:10.3390/md21030186)
Supplement: Supplementary file 1 [file marinedrugs-21-00186-s001.zip › marinedrugs-2235035-supplementary.pdf]

Supplementary Materials

Expanding the Chemical Space of Arsenicin A-C Related Polyarsenicals and Evaluation of Some Analogs as Inhibitors of Glioblastoma Stem Cell Growth

Contents

	Page(s):
**Table S1.** Relevant experimental IR data and calculated frequencies from density functional theory (DFT)-vibrational analysis using the B1B95/6-311+G(3df,2pd) basis set for compounds **8** and **9**.	**S2**
**Figure S1.** ^{1}H- and ^{13}C-NMR spectra of synthetic mixture containing the sulfur nor-adamantane **10-12**.	**S3**
**Figure S2.** EI-MS spectrum of arsenicin D (**13**) present in the HPLC fraction by purification of the polyarsenical mixture from workup of the sponge *E. bargiba*nti extract.	**S4**
**Figure S3.** ^{1}H-NMR spectrum of the natural polyarsenicals mixture from workup of the sponge *E. bargibanti* extract; signals of arsenicin D are highlighted in yellow.	**S4**
**Table S2.** Energies calculated for the structures of arsenicin A-D (**1-3** and **13**) and relative stability of the corresponding structures after O/S replacement by DFT calculation *in vacuo* at B1B95/6-311+G(3df,2pd) level of theory. The results were obtained by applying the following equations:<br>A) $\Delta E = \lvert E\ (\text{Arsenicin X}) - n\ E(\text{S atoms}) + m\ E(\text{O atoms}) - E\ (\text{arsenicin A})\rvert$<br>B) $\Delta E = \lvert E\ (\text{Arsenicin Y}) - m\ E(\text{O atoms}) + n\ E(\text{S atoms}) - E\ (\text{arsenicin B/C/D})\rvert$<br>where X and Y are for B, C or D, n = number of substituted sulfur atoms and m = number of substituted oxygen atoms.	**S5**
**Table S3.** Selectivity Index (SI) values of alkyl polyarsenicals **7-9** and ATO, given by the ratio between the GI_{50} values of non-tumor cell lines and GSC lines, respectively.	**S6**
**Figure S4.** Western blot for p53, p21, and actin in COMI cells treated with 100 μM of camptothecin (CTH) for 16 hours.	**S6**
**Table S4.** GI_{50} values in μM of alkyl polyarsenicals **7-9** and ATO on COMI cells grown either under normoxic conditions (normoxia), under hypoxic conditions for the duration of the treatment (hypoxia 48 h), or under hypoxic conditions for one week prior to drug treatment (hypoxia 1 week).	**S6**
**Figures S5-S11.** Predicted physical-chemical properties and bioavailability radar for compounds **1** and **7-12** evaluated by Swiss-ADME software (http://www.swissadme.ch/ accessed on 9 January 2023).	**S7-S10**
**Figure S12.** WLOGP-versus-TPSA in Brain Or IntestinaL EstimateD permeation method (BOILED)-Egg visualization for alkyl adamantane **7-9** and dimethyl As-S compounds **10-12** in comparison with arsenicin A (**1**) evaluated by using Swiss-ADME software (http://www.swissadme.ch/ accessed on 9 January 2023).	**S10**
**Table S5.** Polar surface area (PSA), lipophilicity (LogP) and blood-brain barrier (BBB) parameters evaluated by using the indicated chemoinformatics software (Swiss-ADME: http://www.swissadme.ch/ accessed on 9 January 2023; Molsft L.L.C.: https://www.molsoft.com/ accessed on 2 March 2023; Molinspiration: https://www.molinspiration.com/ accessed on 2 March 2023).	**S11**
**Figure S13.** ^{1}H- and ^{13}C-NMR spectra of compound **8**.	**S12**
**Figure S14.** ^{1}H- and ^{13}C-NMR spectra of compound **9**.	**S13**
**Table S6.** Seeding numbers (expressed in cells/well) used for testing the alkyl polyarsenicals **7-9** and ATO in 384-well plates.	**S14**

Table S1. Relevant experimental IR data and calculated frequencies from density functional theory (DFT)-vibrational analysis using the B1B95/6-311+G(3df,2pd) basis set for compounds **8** and **9**.

IR frequency (cm^{-1})		Relative Intensity	Assignment
Calculated	Experimental		
	Compound **8**		
463	456	strong	As-O-As bending
727	723	strong	As-C-As stretching
797	795	very strong	As-O stretching
1067	1043	medium	CH twisting
1326	1375	weak	CH_3 symmetric bending
1413	1451	medium	CH_3 twisting
2832	2837	weak	symmetric CH_2 stretching
2867	2843	weak	symmetric CH_3 stretching
2933	2923	weak	asymmetric CH_3 stretching
2941	2954	medium	asymmetric CH_3 stretching
	Compound **9**		
458	443	strong	As-O-As bending
464	465	strong	As-O-As bending
731	717	strong	As-C-As stretching
796	789	very strong	As-O stretching
1072	1063	medium	CH twisting
1418	1461	medium	CH_3 twisting
2820	2827	medium	symmetric CH_2 stretching
2857	2852	medium	symmetric CH_2 stretching
2866	2870	medium	symmetric CH_3 stretching
2894	2916	medium	asymmetric CH_2 stretching
2940	2953	medium	asymmetric CH_3 stretching

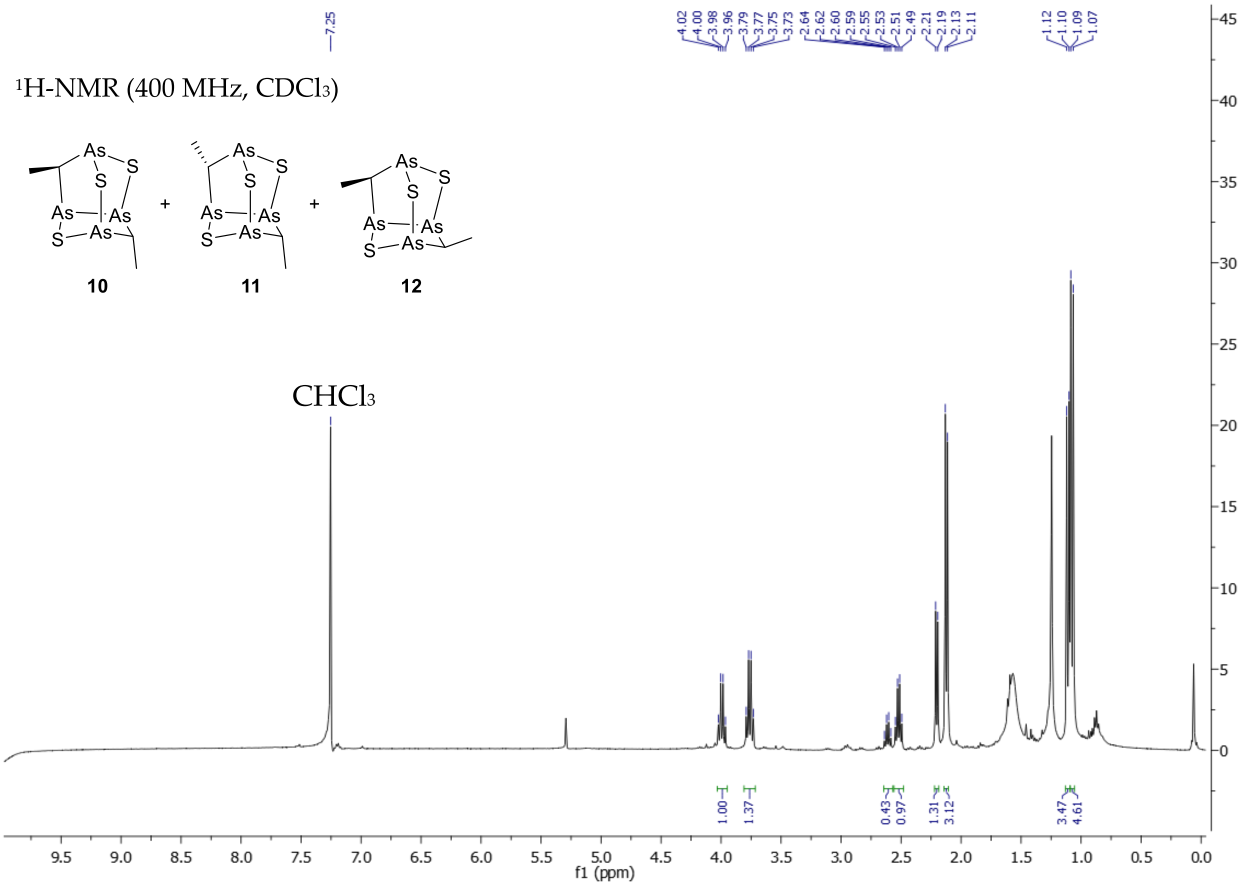

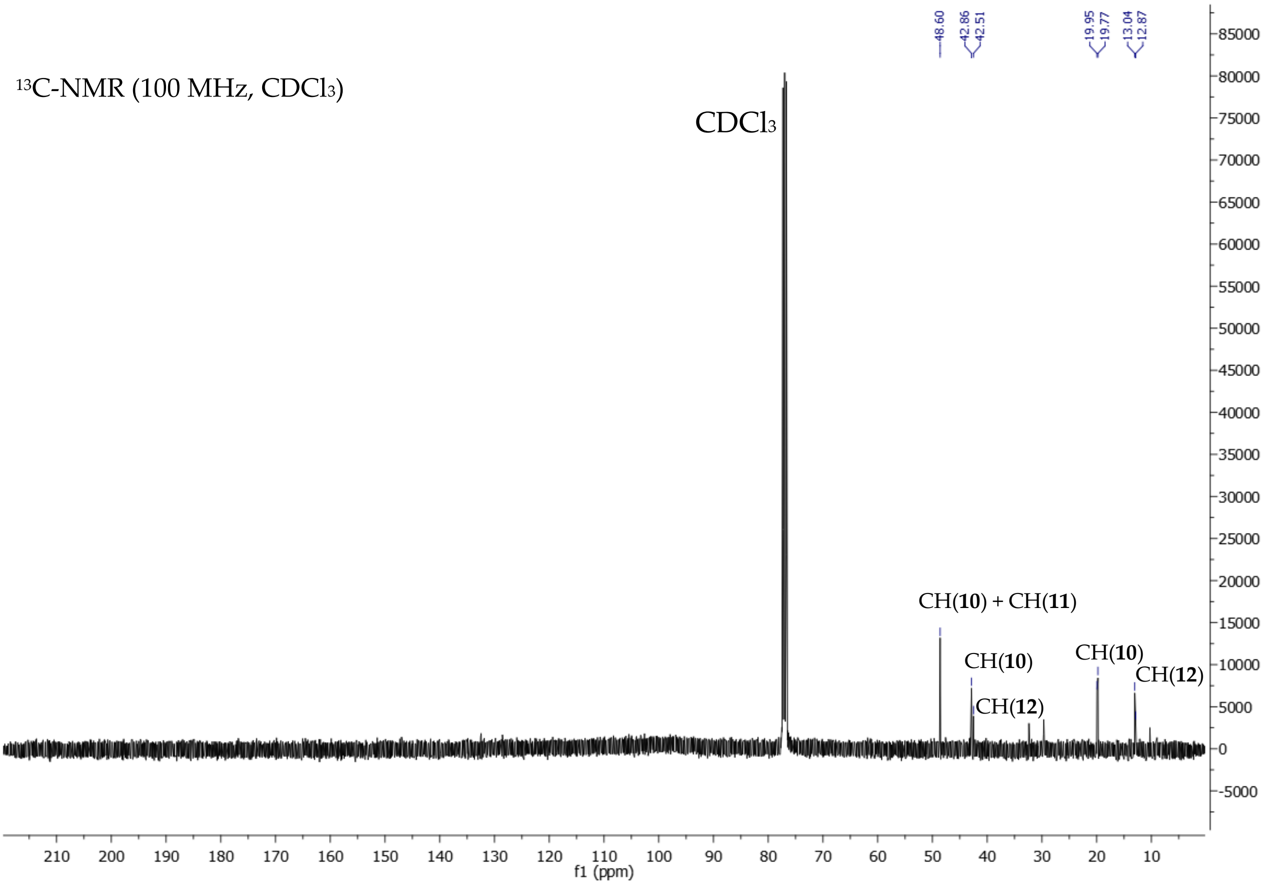

Figure S1. ^{1}H- and ^{13}C-NMR spectra of sulfur derivatives mixture.

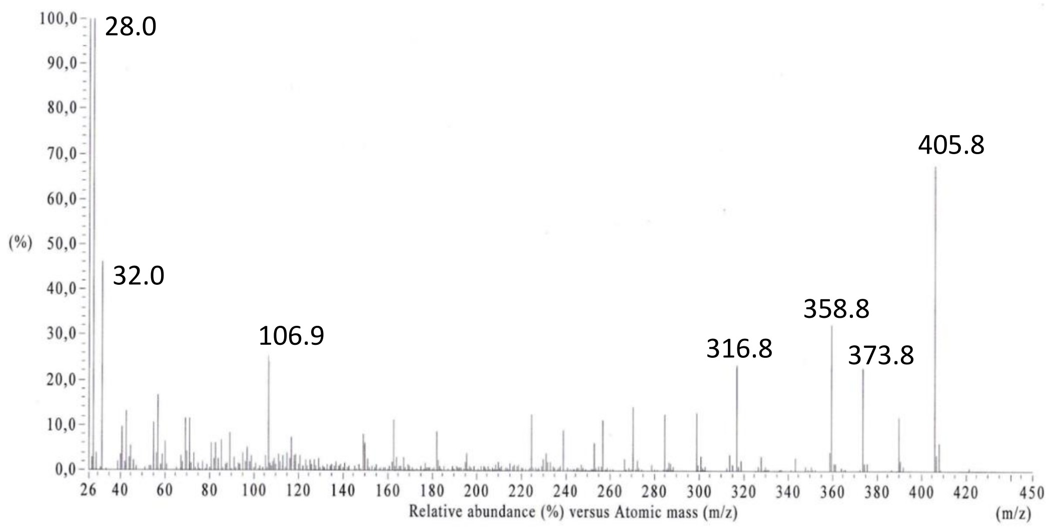

Figure S2. EI-MS spectrum of arsenicin D (**13**) present in the HPLC fraction by purification of the polyarsenical mixture from workup of the sponge *E. bargibanti* extract.

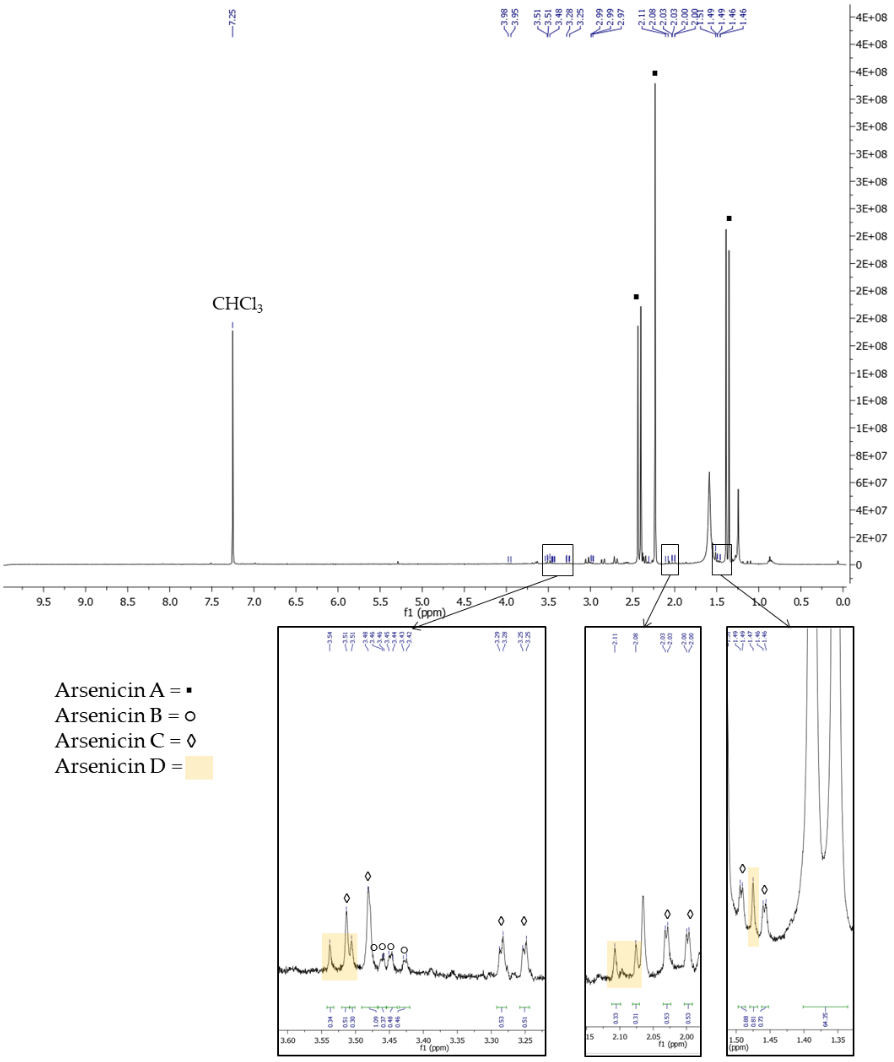

Figure S3. ^{1}H-NMR spectrum of the natural polyarsenicals mixture from workup of the sponge *E. bargibanti* extract; signals of arsenicin D are highlighted in yellow.

Table S2. Energies calculated for the structures of arsenicin A-D (**1**-**3** and **13**) and relative stability of the corresponding structures after O/S replacement by DFT calculation *in vacuo* at B1B95/6-311+G(3df,2pd) level of theory. The results were obtained by applying the following equations:

A) ΔE = ∣E (Arsenicin X) - n E(S atoms) + m E(O atoms) – E (arsenicin A)∣
B) ΔE = ∣E (Arsenicin Y) - m E(O atoms) + n E(S atoms) – E (arsenicin B/C/D)∣

where X and Y are for B, C or D, n = number of substituted sulfur atoms and m = number of substituted oxygen atoms.

	**Equation**	**Energy (a.u.)**	**Δ E (a.u.)**	**Δ E (kcal/mol)**
Arsenicin A (**1**, $C_3H_6As_4O_3$)	A	-9288.336433	0.0	-
Arsenicin B (**2**, $C_3H_6As_4S_2$)	A	-9859.187098	0.451145	285.96
Arsenicin C (**3**, $C_3H_6As_4OS$)	A	-9536.145045	0.35991	227.11
Arsenicin D (**13**, $C_3H_6As_4S_2$)	A	-9859.188891	0.449352	285.27
Arsenicin A (**1**)		-9288.336433	-	-
3S-Arsenicin A ($C_3H_6As_4S_3$)	A	-10257.450842	0.285455	179.13
Arsenicin B (**2**)		-9859.187098		
2O-Arsenicin B ($C_3H_6As_4O_2$)[a]	B	-9213.103230	0.182708	114.70
Arsenicin C (**3**)		-9536.145045		
2O-Arsenicin C ($C_3H_6As_4O_2$)[a]	B	-9213.103230	0.091473	57.40
Arsenicin D (**13**)		-9859.188891		
2O-Arsenicin D($C_3H_6As_4O_2$)	B	-9213.095435	0.17312	108.63
Oxygen atom		-74.964766	-	-
Sulfur atom		-398.098054	-	-

[a] the same structure

Table S3. Selectivity Index (SI) values of alkyl polyarsenicals **7-9** and ATO, given by the ratio between the GI_{50} values of non-tumor cell lines and GSC lines, respectively.

	ATO				7				8				9			
	**ARPE-19**	**MCF10A**	**hTERT-HPNE**	**Hs68**	**ARPE-19**	**MCF10A**	**hTERT-HPNE**	**Hs68**	**ARPE-19**	**MCF10A**	**hTERT-HPNE**	**Hs68**	**ARPE-19**	**MCF10A**	**hTERT-HPNE**	**Hs68**
**COMI**	3.1	1.6	3.1	nd[a]	14.6	16.9	20.8	51.9	17.2	17.8	29.6	124.0	13.5	13.3	43.8	107.3
**VIPI**	0.7	0.3	0.7	nd[a]	1.8	2.1	2.6	6.5	3.9	4.0	6.7	28.2	3.0	2.9	9.7	23.8
**GB6**	1.0	0.5	1.0	nd[a]	2.0	2.3	2.8	7.1	2.5	2.6	4.4	18.2	1.6	1.6	5.1	12.6
**GB7**	2.1	1.1	2.1	nd[a]	24.3	28.2	34.7	86.4	17.2	17.8	29.6	124.0	10.8	10.6	35.0	85.8
**G144**	1.3	0.7	1.3	nd[a]	6.6	7.7	9.5	23.6	9.6	9.9	16.4	68.9	6.8	6.6	21.9	53.6
**G166**	0.7	0.4	0.7	nd[a]	1.5	1.7	2.1	5.3	2.8	2.9	4.8	20.0	2.3	2.2	7.3	17.9
**GB8**	0.8	0.4	0.8	nd[a]	1.6	1.9	2.3	5.8	2.6	2.7	4.5	18.8	1.9	1.9	6.3	15.3
**GSC#1**	1.4	0.7	1.4	nd[a]	3.3	3.8	4.7	11.8	4.5	4.7	7.8	32.6	3.0	2.9	9.7	23.8
**GSC#151**	0.7	0.4	0.7	nd[a]	3.5	4.1	5.0	12.5	7.2	7.4	12.3	51.7	3.2	3.1	10.3	25.2

[a] nd: not determined. SI was not calculated since the GI_{50} value of ATO on Hs68 was greater than the highest concentration tested (>100 μM).

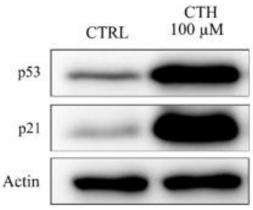

Figure S4. Western blot for p53, p21, and actin in COMI cells treated with 100 μM of camptothecin (CTH) for 16 hours.

Table S4. GI_{50} values[a] in μM of alkyl polyarsenicals **7-9** and ATO on COMI cells grown either under normoxic conditions (normoxia), under hypoxic conditions for the duration of the treatment (hypoxia 48 h), or under hypoxic conditions for one week prior to drug treatment (hypoxia 1 week).

	ATO	7	8	9
**Normoxia**	0.96 ± 0.06	0.15 ± 0.02	0.05 ± 0.07	0.040 ± 0.004
**Hypoxia 48 h**	0.98 ± 0.06	0.15 ± 0.05	0.07 ± 0.02	0.06 ± 0.02
**Hypoxia 1 week**	1.04 ± 0.07	0.14 ± 0.07	0.08 ± 0.04	0.06 ± 0.02

[a] GI_{50} values were calculated upon treating COMI cells with different compound concentrations for 48 h. The GI_{50} values were calculated from n = 6 biological replicates, n = 3 technical replicates each, mean ± SEM.

1

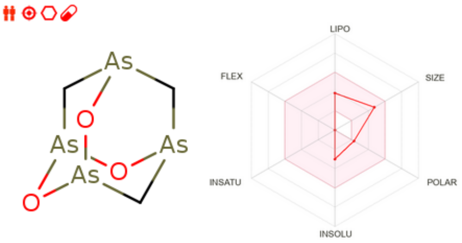

SMILES C1[As]2C[As]3O[As]1C[As](O2)O3

Physicochemical Properties	
Formula	C3H6As4O3
Molecular weight	389.76 g/mol
Num. heavy atoms	10
Num. arom. heavy atoms	0
Fraction Csp3	1.00
Num. rotatable bonds	0
Num. H-bond acceptors	3
Num. H-bond donors	0
Molar Refractivity	40.69
TPSA	27.69 Å²
**Lipophilicity**	
Log $P_{o/w}$ (iLOGP)	0.00
Log $P_{o/w}$ (XLOGP3)	1.21
Log $P_{o/w}$ (WLOGP)	-0.35
Log $P_{o/w}$ (MLOGP)	-1.91
Log $P_{o/w}$ (SILICOS-IT)	-2.12
Consensus Log $P_{o/w}$	-0.63

Water Solubility	
Log *S* (ESOL)	-3.02
Solubility	3.73e-01 mg/ml ; 9.58e-04 mol/l
Class	Soluble
Log *S* (Ali)	-1.39
Solubility	1.59e+01 mg/ml ; 4.09e-02 mol/l
Class	Very soluble
Log *S* (SILICOS-IT)	-0.90
Solubility	4.94e+01 mg/ml ; 1.27e-01 mol/l
Class	Soluble
**Pharmacokinetics**	
GI absorption	High
BBB permeant	No
P-gp substrate	No
CYP1A2 inhibitor	No
CYP2C19 inhibitor	No
CYP2C9 inhibitor	No
CYP2D6 inhibitor	No
CYP3A4 inhibitor	No
Log K_p (skin permeation)	-7.82 cm/s
**Druglikeness**	
Lipinski	Yes; 0 violation
Ghose	No; 1 violation: #atoms<20
Veber	Yes
Egan	Yes
Muegge	No; 1 violation: #C<5
Bioavailability Score	0.55
**Medicinal Chemistry**	
PAINS	0 alert
Brenk	1 alert: heavy_metal
Leadlikeness	No; 1 violation: MW>350
Synthetic accessibility	5.96

Figure S5. Predicted physical-chemical properties and bioavailability radar for arsenicin A (**1**) evaluated by using Swiss-ADME software (http://www.swissadme.ch/ accessed on 9 January 2023).

7

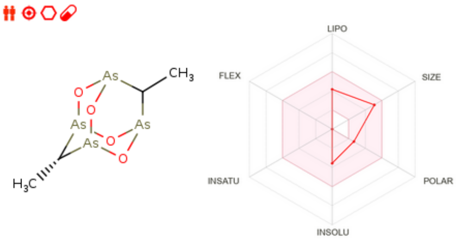

SMILES CC1[As]2O[As]3O[As]1O[As](O2)[C@@H]3C

Physicochemical Properties	
Formula	C4H8As4O4
Molecular weight	419.79 g/mol
Num. heavy atoms	12
Num. arom. heavy atoms	0
Fraction Csp3	1.00
Num. rotatable bonds	0
Num. H-bond acceptors	4
Num. H-bond donors	0
Molar Refractivity	46.58
TPSA	36.92 Å²
**Lipophilicity**	
Log $P_{o/w}$ (iLOGP)	0.00
Log $P_{o/w}$ (XLOGP3)	1.77
Log $P_{o/w}$ (WLOGP)	-0.10
Log $P_{o/w}$ (MLOGP)	-2.30
Log $P_{o/w}$ (SILICOS-IT)	-2.94
Consensus Log $P_{o/w}$	-0.71

Water Solubility	
Log *S* (ESOL)	-3.56
Solubility	1.16e-01 mg/ml ; 2.77e-04 mol/l
Class	Soluble
Log *S* (Ali)	-2.16
Solubility	2.88e+00 mg/ml ; 6.87e-03 mol/l
Class	Soluble
Log *S* (SILICOS-IT)	-0.65
Solubility	9.51e+01 mg/ml ; 2.26e-01 mol/l
Class	Soluble
**Pharmacokinetics**	
GI absorption	High
BBB permeant	No
P-gp substrate	No
CYP1A2 inhibitor	No
CYP2C19 inhibitor	No
CYP2C9 inhibitor	No
CYP2D6 inhibitor	No
CYP3A4 inhibitor	No
Log K_p (skin permeation)	-7.60 cm/s
**Druglikeness**	
Lipinski	Yes; 0 violation
Ghose	Yes
Veber	Yes
Egan	Yes
Muegge	No; 1 violation: #C<5
Bioavailability Score	0.55
**Medicinal Chemistry**	
PAINS	0 alert
Brenk	1 alert: heavy_metal
Leadlikeness	No; 1 violation: MW>350
Synthetic accessibility	6.32

Figure S6. Predicted physical-chemical properties and bioavailability radar for compounds **7** evaluated by using Swiss-ADME software (http://www.swissadme.ch/ accessed on 9 January 2023).

8

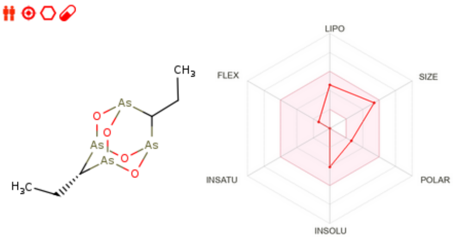

SMILES CCC1[As]2O[As]3O[As]1O[As](O2)[C@@H]3CC

Physicochemical Properties	
Formula	C6H12As4O4
Molecular weight	447.84 g/mol
Num. heavy atoms	14
Num. arom. heavy atoms	0
Fraction Csp3	1.00
Num. rotatable bonds	2
Num. H-bond acceptors	4
Num. H-bond donors	0
Molar Refractivity	56.20
TPSA	36.92 Å²

Lipophilicity	
Log $P_{o/w}$ (iLOGP)	0.00
Log $P_{o/w}$ (XLOGP3)	2.49
Log $P_{o/w}$ (WLOGP)	0.69
Log $P_{o/w}$ (MLOGP)	-1.52
Log $P_{o/w}$ (SILICOS-IT)	-2.62
Consensus Log $P_{o/w}$	-0.19

Water Solubility	
Log *S* (ESOL)	-4.05
Solubility	3.96e-02 mg/ml ; 8.84e-05 mol/l
Class	Moderately soluble
Log *S* (Ali)	-2.91
Solubility	5.50e-01 mg/ml ; 1.23e-03 mol/l
Class	Soluble
Log *S* (SILICOS-IT)	-1.43
Solubility	1.67e+01 mg/ml ; 3.73e-02 mol/l
Class	Soluble

Pharmacokinetics	
GI absorption	High
BBB permeant	Yes
P-gp substrate	No
CYP1A2 inhibitor	No
CYP2C19 inhibitor	No
CYP2C9 inhibitor	No
CYP2D6 inhibitor	No
CYP3A4 inhibitor	No
Log K_p (skin permeation)	-7.26 cm/s

Druglikeness	
Lipinski	Yes; 0 violation
Ghose	Yes
Veber	Yes
Egan	Yes
Muegge	Yes
Bioavailability Score	0.55

Medicinal Chemistry	
PAINS	0 alert
Brenk	1 alert: heavy_metal
Leadlikeness	No; 1 violation: MW>350
Synthetic accessibility	6.41

Figure S7. Predicted physical-chemical properties and bioavailability radar for compounds **8** evaluated by using Swiss-ADME software (http://www.swissadme.ch/ accessed on 9 January 2023).

9

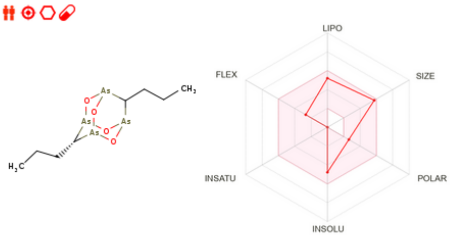

SMILES CCCC1[As]2O[As]3O[As]1O[As](O2)[C@@H]3CCC

Physicochemical Properties	
Formula	C8H16As4O4
Molecular weight	475.90 g/mol
Num. heavy atoms	16
Num. arom. heavy atoms	0
Fraction Csp3	1.00
Num. rotatable bonds	4
Num. H-bond acceptors	4
Num. H-bond donors	0
Molar Refractivity	65.81
TPSA	36.92 Å²

Lipophilicity	
Log $P_{o/w}$ (iLOGP)	0.00
Log $P_{o/w}$ (XLOGP3)	3.57
Log $P_{o/w}$ (WLOGP)	1.47
Log $P_{o/w}$ (MLOGP)	-0.83
Log $P_{o/w}$ (SILICOS-IT)	-2.06
Consensus Log $P_{o/w}$	0.43

Water Solubility	
Log *S* (ESOL)	-4.78
Solubility	7.98e-03 mg/ml ; 1.68e-05 mol/l
Class	Moderately soluble
Log *S* (Ali)	-4.03
Solubility	4.43e-02 mg/ml ; 9.31e-05 mol/l
Class	Moderately soluble
Log *S* (SILICOS-IT)	-2.21
Solubility	2.95e+00 mg/ml ; 6.19e-03 mol/l
Class	Soluble

Pharmacokinetics	
GI absorption	High
BBB permeant	Yes
P-gp substrate	No
CYP1A2 inhibitor	No
CYP2C19 inhibitor	No
CYP2C9 inhibitor	No
CYP2D6 inhibitor	No
CYP3A4 inhibitor	No
Log K_p (skin permeation)	-6.67 cm/s

Druglikeness	
Lipinski	Yes; 0 violation
Ghose	Yes
Veber	Yes
Egan	Yes
Muegge	Yes
Bioavailability Score	0.55

Medicinal Chemistry	
PAINS	0 alert
Brenk	1 alert: heavy_metal
Leadlikeness	No; 2 violations: MW>350, XLOGP3>3.5
Synthetic accessibility	6.53

Figure S8. Predicted physical-chemical properties and bioavailability radar for compounds **9** evaluated by using Swiss-ADME software (http://www.swissadme.ch/ accessed on 9 January 2023).

10

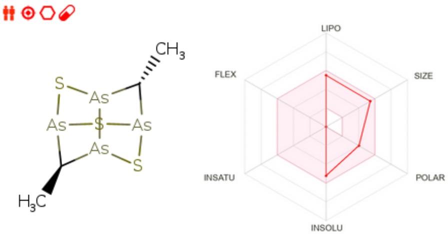

SMILES C[C@@H]1[As]2S[As]3S[As]1[As](S2)[C@H]3C

Physicochemical Properties	
Formula	C4H8As4S3
Molecular weight	451.99 g/mol
Num. heavy atoms	11
Num. arom. heavy atoms	0
Fraction Csp3	1.00
Num. rotatable bonds	0
Num. H-bond acceptors	0
Num. H-bond donors	0
Molar Refractivity	65.02
TPSA	75.90 Å²

Lipophilicity	
Log $P_{o/w}$ (iLOGP)	0.00
Log $P_{o/w}$ (XLOGP3)	4.12
Log $P_{o/w}$ (WLOGP)	2.12
Log $P_{o/w}$ (MLOGP)	1.42
Log $P_{o/w}$ (SILICOS-IT)	-0.98
Consensus Log $P_{o/w}$	1.34

Water Solubility	
Log *S* (ESOL)	-5.24
Solubility	2.61e-03 mg/ml ; 5.78e-06 mol/l
Class	Moderately soluble
Log *S* (Ali)	-5.42
Solubility	1.72e-03 mg/ml ; 3.80e-06 mol/l
Class	Moderately soluble
Log *S* (SILICOS-IT)	-1.98
Solubility	4.69e+00 mg/ml ; 1.04e-02 mol/l
Class	Soluble

Pharmacokinetics	
GI absorption	High
BBB permeant	Yes
P-gp substrate	No
CYP1A2 inhibitor	No
CYP2C19 inhibitor	No
CYP2C9 inhibitor	No
CYP2D6 inhibitor	No
CYP3A4 inhibitor	No
Log K_p (skin permeation)	-6.13 cm/s

Druglikeness	
Lipinski	Yes; 0 violation
Ghose	No; 1 violation: #atoms<20
Veber	Yes
Egan	Yes
Muegge	No; 1 violation: #C<5
Bioavailability Score	0.55

Medicinal Chemistry	
PAINS	0 alert
Brenk	1 alert: heavy_metal
Leadlikeness	No; 2 violations: MW>350, XLOGP3>3.5
Synthetic accessibility	6.09

Figure S9. Predicted physical-chemical properties and bioavailability radar for compounds **10** evaluated by using Swiss-ADME software (http://www.swissadme.ch/ accessed on 9 January 2023).

11

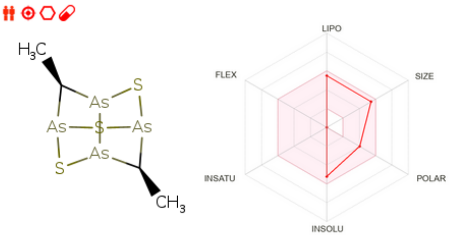

SMILES C[C@@H]1[As]2S[As]3S[As]1[As](S2)[C@@H]3C

Physicochemical Properties	
Formula	C4H8As4S3
Molecular weight	451.99 g/mol
Num. heavy atoms	11
Num. arom. heavy atoms	0
Fraction Csp3	1.00
Num. rotatable bonds	0
Num. H-bond acceptors	0
Num. H-bond donors	0
Molar Refractivity	65.02
TPSA	75.90 Å²

Lipophilicity	
Log $P_{o/w}$ (iLOGP)	0.00
Log $P_{o/w}$ (XLOGP3)	4.12
Log $P_{o/w}$ (WLOGP)	2.12
Log $P_{o/w}$ (MLOGP)	1.42
Log $P_{o/w}$ (SILICOS-IT)	-0.98
Consensus Log $P_{o/w}$	1.34

Water Solubility	
Log *S* (ESOL)	-5.24
Solubility	2.61e-03 mg/ml ; 5.78e-06 mol/l
Class	Moderately soluble
Log *S* (Ali)	-5.42
Solubility	1.72e-03 mg/ml ; 3.80e-06 mol/l
Class	Moderately soluble
Log *S* (SILICOS-IT)	-1.98
Solubility	4.69e+00 mg/ml ; 1.04e-02 mol/l
Class	Soluble

Pharmacokinetics	
GI absorption	High
BBB permeant	Yes
P-gp substrate	No
CYP1A2 inhibitor	No
CYP2C19 inhibitor	No
CYP2C9 inhibitor	No
CYP2D6 inhibitor	No
CYP3A4 inhibitor	No
Log K_p (skin permeation)	-6.13 cm/s

Druglikeness	
Lipinski	Yes; 0 violation
Ghose	No; 1 violation: #atoms<20
Veber	Yes
Egan	Yes
Muegge	No; 1 violation: #C<5
Bioavailability Score	0.55

Medicinal Chemistry	
PAINS	0 alert
Brenk	1 alert: heavy_metal
Leadlikeness	No; 2 violations: MW>350, XLOGP3>3.5
Synthetic accessibility	6.09

Figure S10. Predicted physical-chemical properties and bioavailability radar for compounds **11** evaluated by using Swiss-ADME software (http://www.swissadme.ch/ accessed on 9 January 2023).

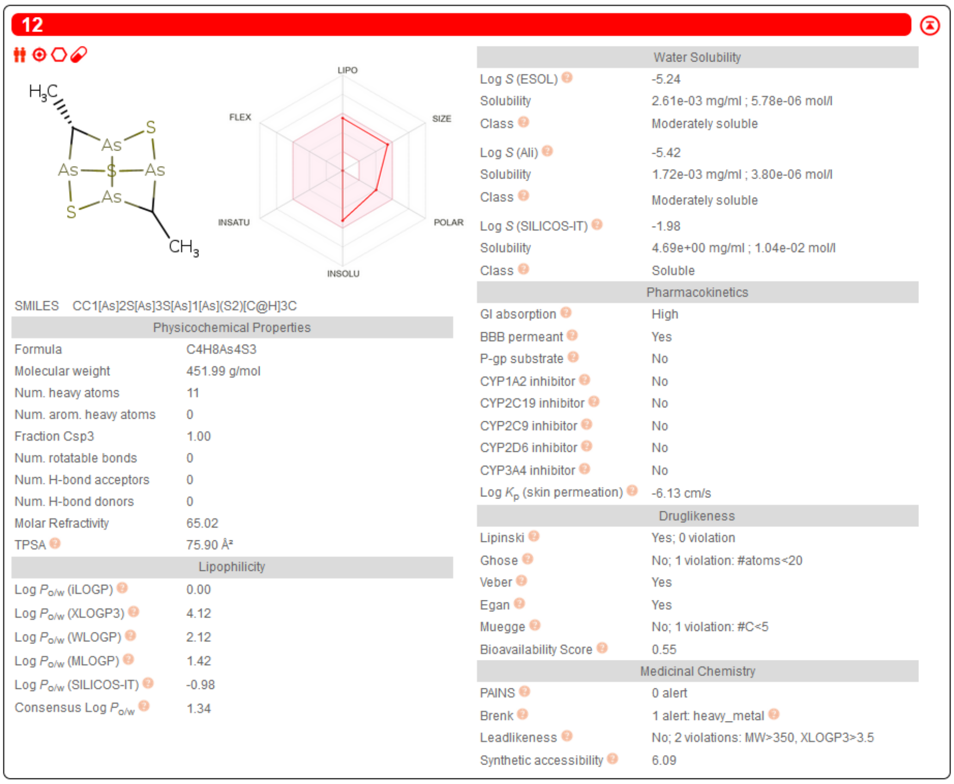

Figure S11. Predicted physical-chemical properties and bioavailability radar for compounds **12** evaluated by using Swiss-ADME software (http://www.swissadme.ch/ accessed on 9 January 2023).

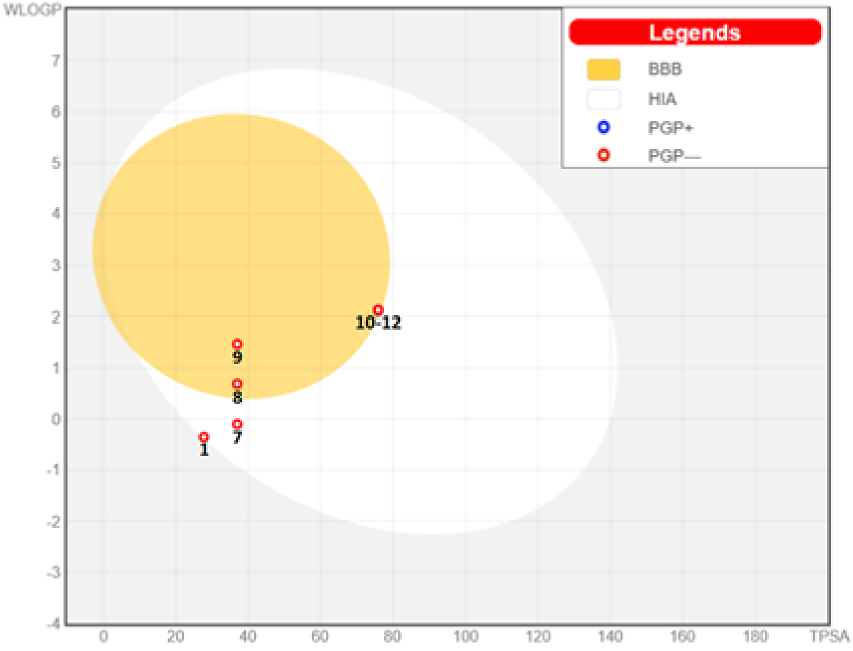

Figure S12. WLOGP-versus-TPSA in Brain Or IntestinaL EstimateD permeation method (BOILED)-Egg visualization for alkyl adamantane **7-9** and dimethyl As-S compounds **10-12** in comparison with arsenicin A (**1**) evaluated by using Swiss-ADME software (http://www.swissadme.ch/ accessed on 9 January 2023).

Table S5. Polar surface area (PSA), lipophilicity (LogP) and blood-brain barrier (BBB) parameters evaluated by using the indicated chemoinformatics software (Swiss-ADME: http://www.swissadme.ch/ accessed on 9 January 2023; Molsft L.L.C.: https://www.molsoft.com/ accessed on 2 March 2023; Molinspiration: https://www.molinspiration.com/ accessed on 2 March 2023).

Web tool	Properties	Arsenicin A	Compound 7	Compound 8	Compound 9
**SwissADME**	TPSA (Å^2)	27.69	36.92	36.92	36.92
	WLogP	-0.35	-0.10	0.69	1.47
	Consensus Log P	-0.63	-0.71	-0.19	0.43
	BBB permeant	No	No	Yes	Yes
**Molinspiration**	TPSA (Å^2)	27.70	36.94	36.94	36.94
	miLog P	-3.03	-2.36	-1.35	-0.23
	BBB	-	-	-	-
**Molsoft L.L.C.**	MolPSA (Å^2)	44.01	42.38	42.38	42.38
	MolLog P	-0.34	0.06	1.03	1.98
	BBB score[a]	3.77	4.03	4.12	4.19

[a] BBB Score: 6-High, 0-Low

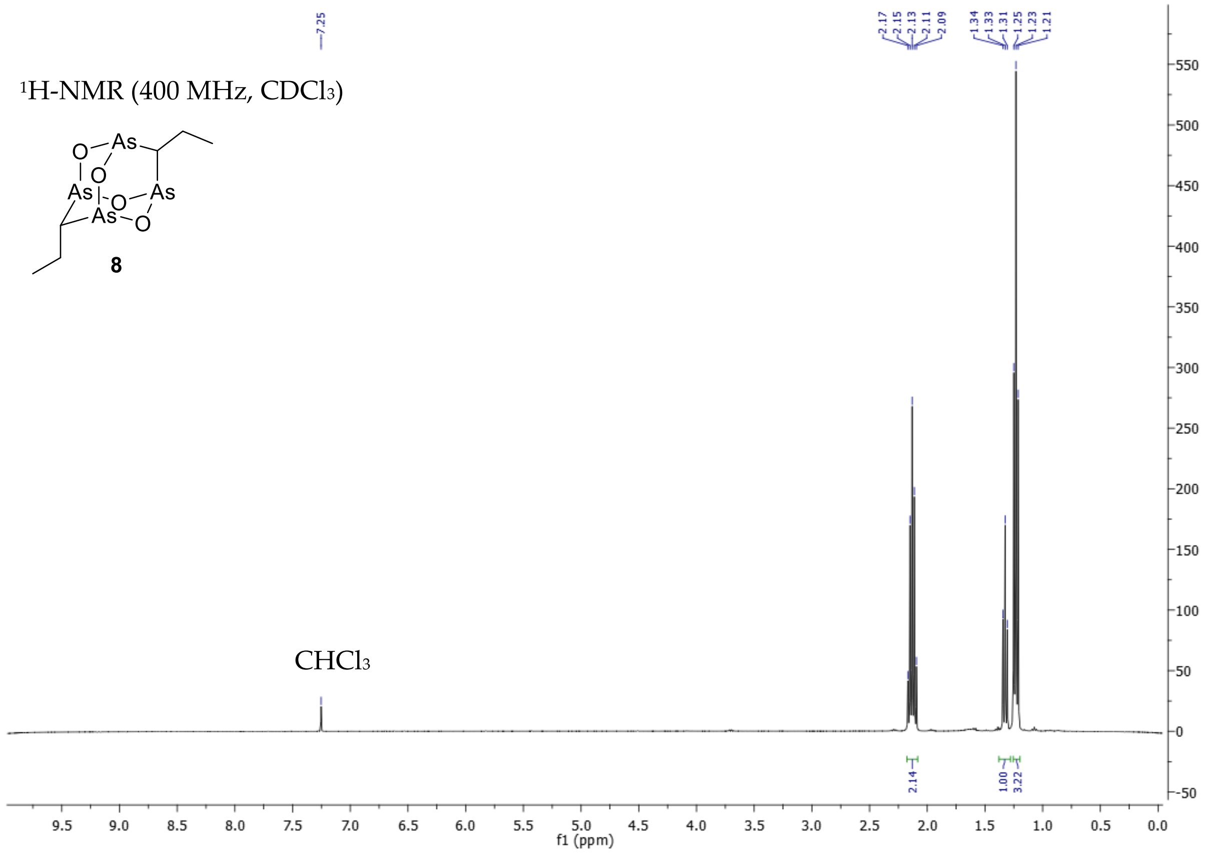

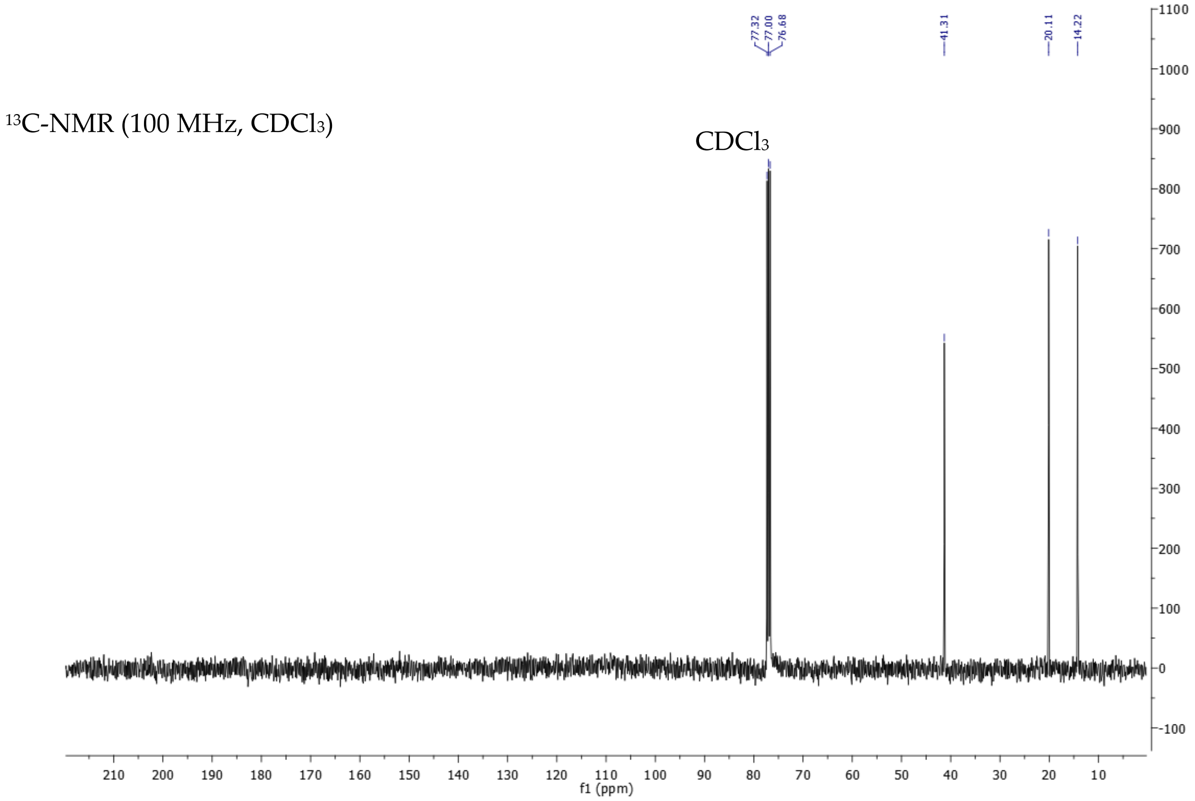

Figure S13. ¹H- and ¹³C-NMR spectra of compound **8**.

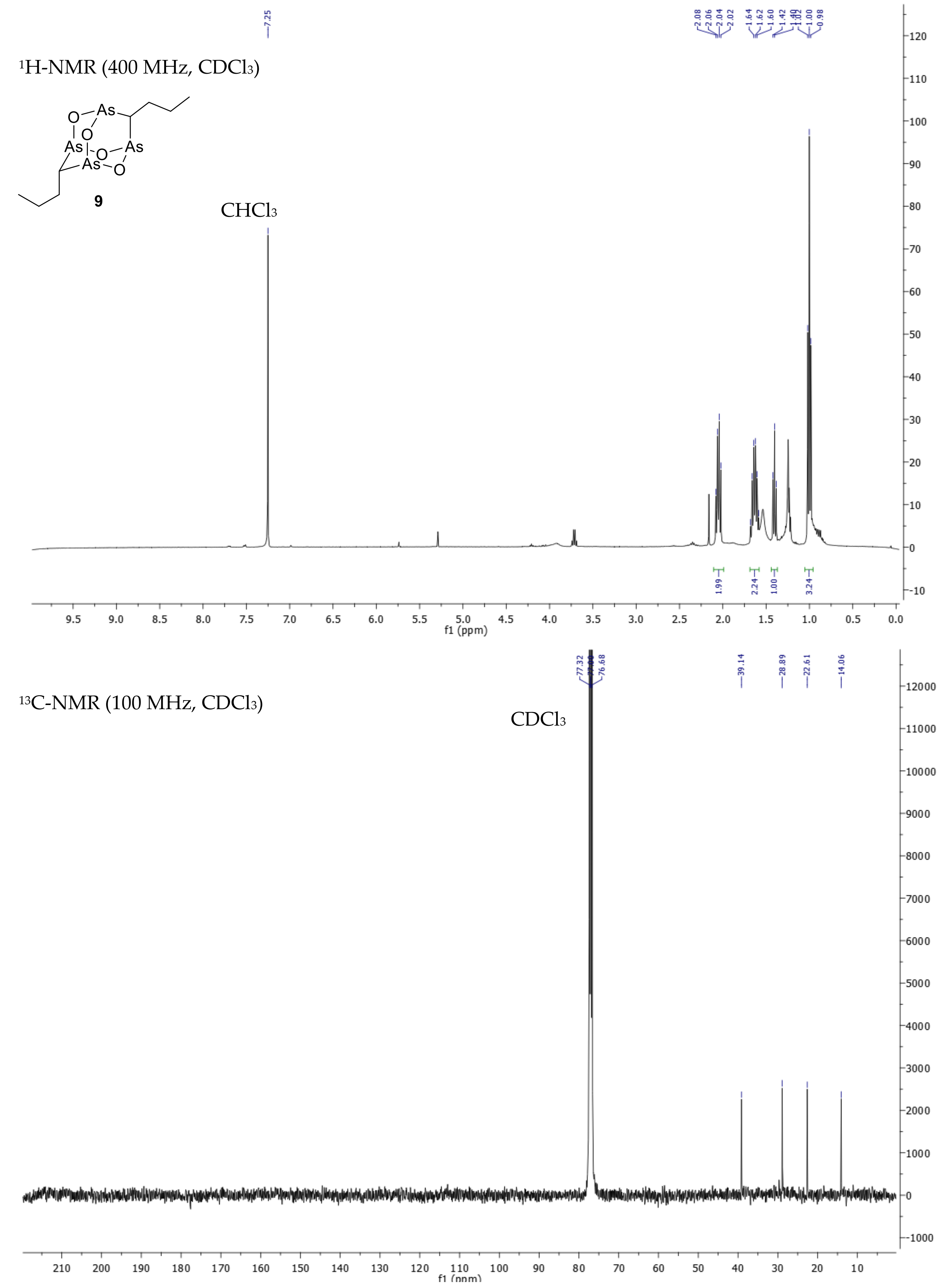

Figure S14. ^{1}H- and ^{13}C-NMR spectra of compound **9**.

Table S6. Seeding numbers (expressed in cells/well) used for testing the alkyl polyarsenicals **7-9** and ATO in 384-well plates.

Cell Line	Seeding Number (cells/well)
COMI	1000
VIPI	1000
GB6	3000
GB7	3000
G144	2000
G166	1000
GB8	1500
GSC#1	1500
GSC#151	1500
ARPE-19	1500
MCF10A	1000
hTERT-HPNE	1500
Hs68	1500
